# Effects of a multicomponent training and a detraining period on cognitive and functional performance of older adults at risk of frailty

**DOI:** 10.1007/s40520-025-03011-w

**Published:** 2025-04-07

**Authors:** Ana Moradell, Isabel Iguacel, David Navarrete-Villanueva, Ángel Iván Fernández-García, Marcela González-Gross, Jorge Pérez-Gómez, Ignacio Ara, Jose Antonio Casajús, Alba Gómez-Cabello, Germán Vicente-Rodríguez

**Affiliations:** 1 Growth, Exercise, Nutrition and Development (EXER-GENUD) Research Group, Universidad de Zaragoza, Zaragoza, Spain; 2 Exercise and Health Spanish Research Net (EXERNET) (RED2022-134800-T),, Zaragoza, Spain; 3 Instituto de investigación Sanitaria de Aragón (IIS Aragón), Zaragoza, 50009 Spain; 4 Instituto Agroalimentario de Aragón-IA2 (Universidad de Zaragoza-CITA), Zaragoza, 50090 Spain; 5 Centro de Investigación Biomédica en Red de Fisiopatología de la Obesidad y Nutrición (CIBERObn), Madrid, Spain; 6 Department of Physiatry and Nursing, Faculty of Health, University of Zaragoza, Zaragoza, 50009 Spain; 7 Department of Physiatry and Nursing, Faculty of Health and Sport Sciences, University of Zaragoza, Zaragoza, 50009 Spain; 8 ImFINE Research Group, Universidad Politécnica de Madrid, Madrid, 28040 Spain; 9 HEME Research Group, University of Extremadura, Cáceres, 10003 Spain; 10 GENUD-Toledo Research Group, Universidad de Castilla-La Mancha, Toledo, 45071 Spain; 11 CIBER en Fragilidad y Envejecimiento Saludable (CIBERFES), Madrid, Spain; 12 Department of Physiatry and Nursing, Faculty of Medicine, University of Zaragoza, Zaragoza, 50009 Spain; 13 Centro Universitario de la Defensa- Academia General Militar, Zaragoza, 50090 Spain; 14 Planta Edificio SAI (Servicio de Apoyo a la Investigación), Universidad de Zaragoza, C/Pedro Cerbuna nº 13, Zaragoza, 50013 Spain

**Keywords:** Ageing, Dementia, Physical exercise, Cognition, Dual tasks

## Abstract

**Aims:**

This study analyzes the effects of a 6-month multicomponent exercise program (MCT) followed by a 4-month detraining period on functional and cognitive status in pre-frail and frail older adults.

**Methods:**

A total of 108 pre-frail and frail adults aged 65 and older participated in the study. They were assigned by convenience to either a control group (CG) or an intervention group (IG). The IG underwent a 6-month MCT followed by a 4-month detraining period. Assessments included a DT test, the Timed Up and Go (TUG) test, the Mini-Mental State Examination (MMSE), and evaluations of basic and instrumental activities of daily living. Data were analyzed using repeated-measures ANOVA.

**Results:**

Significant group-by-time interactions were observed for the DT test (*p* < 0.05). The IG showed improved DT performance after the 6-month MCT (4.0, 95% CI: 2.2 to 5.7 s), followed by a decline after the detraining period (-1.1, 95% CI: -2.1 to -0.2 s). However, performance after detraining remained higher than at baseline (2.9, 95% CI: 1.0 to 4.6 s, *p* < 0.05). No statistically significant changes were observed in the CG. Additionally, no significant effects were found for MMSE scores or daily activity questionnaires.

**Conclusion:**

MCT had beneficial effects on functional and cognitive performance in older adults, as assessed by the DT test. However, improvements in DT performance did not translate into better daily life activities. Although the 4-month detraining period negatively impacted DT performance, the results remained superior to baseline levels.

**Trial registration number:**

NCT03831841 and date of registration: 5th of November 2018.

## Introduction

In the last 50 years, the population aged over 60 has tripled, and by 2050, this group is expected to represent 25% of the world’s population [1]. Frailty is one of the most problematic manifestations of population ageing, as it is a multifactorial syndrome that makes older adults vulnerable to adverse events such as falls, disabilities, or illnesses that may require hospitalization [2].

Frailty is characterized by a decline in physical function, which often—but not always—coincides with cognitive deterioration as ageing progresses [3]. Although these declines can occur independently, they frequently interact, compounding the vulnerability of older adults. Walking while talking is a common motor-cognitive task in daily life, and dual-task performance is a reliable predictor of cognitive impairment over time in healthy older adults [4] and a risk factor for other adverse events such as falls [5]. Unlike single-task assessments, which may not fully capture the complexity of real-life functional challenges, dual-task evaluations provide a more dynamic measure of cognitive-motor interaction, revealing deficits that might otherwise go unnoticed. Moreover, its decline begins to manifest in the middle of the sixth decade of life, even when usual gait performance remains unchanged with age in healthy middle-aged adults [6]. Given the importance of assessing physical and cognitive impairments simultaneously, dual-task (DT) tests, which involve performing a motor task alongside a cognitive task [7], have proven useful.

Exercise in aging, particularly for frailty, is an effective strategy for maintaining functional capacity, which provides individuals with autonomy and independence in performing daily activities and enhances cognition in older adults [8]. In particular, multicomponent training (MCT) appears to be, to date, the most effective program for improving physical functionality [9, 10]. MCT includes several skills and components in one training session; muscular strength aerobic capacity, flexibility, coordination, agility, etc [11]. Although the benefits of MCT have been extensively studied in relation to physical function, and there is evidence supporting potential benefits for cognitive performance [12], less is known about its effects on older adults at risk of frailty, particularly those without cognitive impairment and in their ability to perform dual-task activities. Their frailty status or low functional capacity, combined with a relatively preserved cognitive state, may suggest that the primary benefits of MCT in this population would be more pronounced in physical function rather than cognitive performance [13, 14]. This necessitates further research into the benefits of different types of training in later life. Moreover, by targeting DT activities, this research could provide insights into interventions that may reduce the risk of falls and improve overall mobility in older pre-frail and frail individuals. Increased knowledge of these non-pharmacological interventions that improve the quality of life in later life would help prevent adverse age-related situations, reduce healthcare costs, and prevent older adults from becoming dependent [9].

Furthermore, despite the well-established beneficial effects of exercise programs on older adults’ physical and cognitive health, the periods when these activities cease—such as during summer holidays—should not be neglected. When implementing an exercise program, it is necessary to consider the consequences of a detraining period on the beneficial effects of physical exercise on the health of older adults [15].

Therefore, the main aim of this study was to investigate whether a 6-month MCT prevents the loss of functional and cognitive capacity in pre-frail and frail older adults and to determine whether a 4-month detraining period affects the benefits derived from the MCT intervention. In addition, daily activity performance and cognitive status changes were studied separately.

## Materials and methods

The present study was designed as an intervention study. The sample for this study is part of the EXERNET-Elder 3.0 project, which was developed to assess the benefits and effects of a 6-month exercise intervention program followed by a 4-month detraining period. The program was based on current scientific evidence regarding frailty, physical fitness, body composition, social relationships, and health-related quality of life. The total duration of the program was 10 months, running from June 2018 to November 2019 [16].

### Participants

**Fig. 1:**
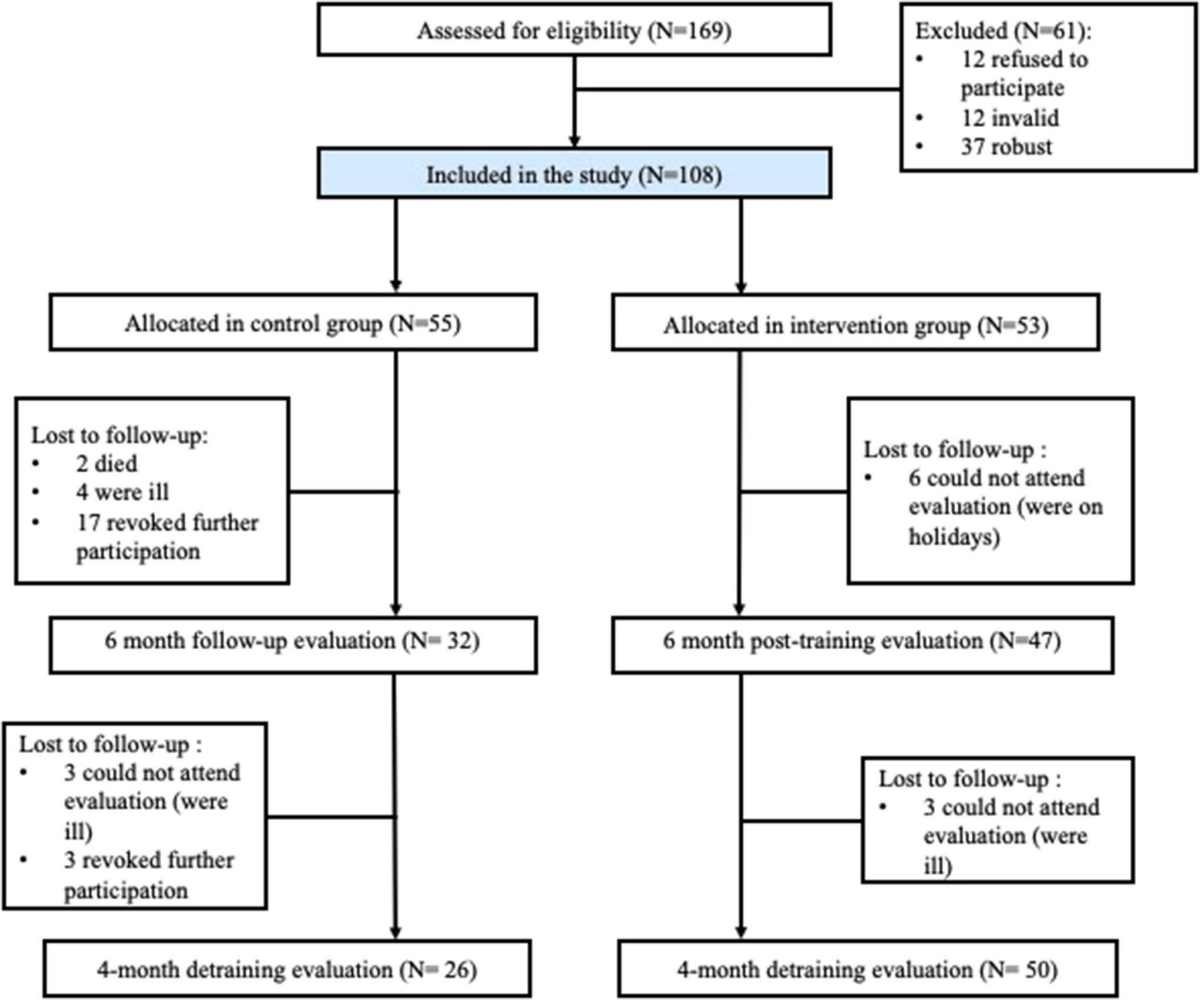
Participants enrolment

Participants were selected in the city of Zaragoza (Spain) from the users of four primary care health centers and three nursing homes. Doctors, nurses, and managers of these health centers selected potential participants aged 65 and older to be assessed using the Short Physical Performance Battery (SPPB) [17]. The tests included in this battery are explained in detail below. Only those who scored between 4 and 9 points (inclusive), classified as pre-frail and frail, were eligible for inclusion in the project. The exclusion criteria were a diagnosis of dementia and/or cancer. The final sample included 108 participants (31 men and 77 women, 71.3%). The age of the participants ranged from 68 to 94 years. Figure 1 shows the participant enrollment process.

Participants were divided into a control group (CG), which continued their usual lifestyle and routine activities throughout the program, and an intervention group (IG). This division was made for convenience, based on the preferences and availability of the older participants, to maximize participation. The IG participated in the MCT training 3 days a week for 6 months, followed by a 4-month period during which MCT was interrupted, and participants returned to their usual lifestyle. Both CG and IG were assessed at three different time points: first, before the start of the MCT; second, at the end of the MCT; and third, at the end of the 4-month detraining period. Additionally, four health-related speeches on healthy aging were offered throughout the 6-month period to ensure CG attendance. These speeches specifically covered frailty, sarcopenia, physical exercise in older adults, and nutrition.

Before the intervention began, all older adults in the study agreed to participate voluntarily and signed an informed consent form after receiving detailed information about the purpose, procedures, benefits, and risks of the study. This information was given both verbally and in writing.

### Multicomponent exercise intervention

The Elder-fit MCT was a six-month physical exercise program with three one-hour sessions weekly [16]. It was designed according to the principles of individualization, periodization, and progression [18]. Training groups consisted of 10–12 participants and one specialized instructor per group. Most of the exercises were performed in pairs, combining participants with lower and higher functional capacity to encourage mutual support.

In general, the exercise program consisted of sessions targeting different fitness components. Two of the three weekly sessions focused on strength exercises, while the third one was based on endurance activities. However, this main part included exercises that engaged multiple fitness components, such as dynamic balance, coordination, and functional tasks related to daily life activities. The sessions were spaced 48 h apart, with strength sessions on Mondays and Fridays and endurance sessions on Wednesdays. Each session was structured to include a 10-minute warm-up, 35–40 min of the main exercise, and 10–15 min of cool-down. Participants performed joint mobility, balance, and cardiorespiratory exercises during the warm-up. The cool-down mainly consisted of stretching exercises. In the last five minutes of the session, after the cool-down, participants were asked to perform cognitive tasks to improve memorization, calculation, orientation, language, reasoning, and executive function. An occupational therapist specializing in cognitive training designed this final part, as described in Table 1.

**Table 1:** Cognitive aspects, sessions conducted, and types of oral cognitive stimulation exercises performed in the multicomponent training program

Cognitive Aspects	Sessions	Total Sessions	Types of Oral Cognitive Stimulation Exercises
Memory	3, 9, 15, 21, 27, 29, 33, 39, 45, 51	10	- Immediate repetition
			- Categorization
			- Immediate memory
			- Short-term memory
			- Long-term memory
Language	4, 7, 10, 13, 16, 17, 22, 25, 28, 31, 34, 37, 40, 43, 46, 49, 52, 55	18	- Verbal fluency: words starting with a letter or syllable
			- Word association
			- Popular sayings
			- Synonyms and antonyms
Executive Functions	5, 11, 23, 35, 41, 47	6	- Numerical codes
			- Word association
			- Social skills
			- Tasks related to daily life activities
Reasoning	6, 12, 18, 19, 24, 30, 36, 42, 48, 53	10	- Logical solutions
			- Object properties
			- Riddles
			- Completing logical sentences
Calculation	8, 9, 13, 14, 15, 17, 20, 21, 24, 26, 27, 29, 32, 38, 44, 50, 56	17	- Arithmetic operations: addition, subtraction
			- Seriation
			- Discussing the numbers forming different figures
Temporal and Spatial Orientation	3, 4, 7, 8, 33, 35, 36, 39, 40, 41, 42, 48, 50, 53	14	- Temporal orientation: recalling the date (day of the week, day of the month, month, year, and season)
			- Spatial orientation: recalling the address of their location (street, number, floor, city, province, and country)

The planning of the exercise sessions was divided into four phases, each with a specific objective and standardized framework. The organization and progression of these sessions are explained in more detail in the tables of the articles published by Fernández-García A.I. et al. [16, 19]. Most of the exercises are performed in pairs, combining participants with lower and higher functional capacity to encourage mutual support. However, training loads were adapted to each participant’s functional capacity, ensuring they could perform the exercises independently. The instructor’s role was to provide corrections and assistance when it was necessary. Additionally, exercises with a higher risk of falls, such as balance training, were performed with supports or close to them to ensure safety.

### Physical and cognitive status and dual-task performance measurements

To standardize the process, all the three evaluations were performed in the same order: anthropometric measurements, questionnaires (including cognitive assessment), and physical fitness tests (including the DT test). The main variables evaluated in the project are detailed below.

The SPPB was used to assess the physical function of the participants. This tool consists of three short tests; 5-sit-to-stand, 4-metre walking speed and the Romberg balance test [20]. Based on the total score obtained, participants were classified as frail (scoring between 4 and 6) or pre-frail (scoring between 7 and 9) [21].

Additionally, nine test, modified from the Eurofit [22] and Senior fitness [23] tests batteries, were used to assess physical fitness. The tests were administered as follows: Static balance (Flamingo Test), upper extremity strength (Arm Curl Test), lower extremity strength (Chair Stand Test), upper extremity flexibility (Back Scratch Test), lower extremity flexibility (Chair Sit-and-Reach Test), agility/dynamic balance (8-foot time-up and go test (TUG)), maximum walking speed (30-m walk test), aerobic capacity (6-minute walk test) and maximum handgrip strength (Takei TKK 5401, Tokyo, Japan).

The Mini Mental State Examination (MMSE) questionnaire was administered to assess the cognitive level of the participants. It consists of eleven items (temporal orientation, spatial orientation, fixation, calculation, memory, nomination, repetition, comprehension, reading, writing and drawing). This test offers a total of 30 points, with a score > 27 indicating a good cognitive level. A score between 25 and 27 may indicate mild cognitive impairment, while a score ≤ 24 indicates mild or moderate cognitive impairment [24, 25, 26].

Furthermore, a DT test was developed. The DT generally consists of performing functional and cognitive tasks simultaneously [7, 27]. This instrument aims to test the brain’s ability to perform previously learned movements, such as walking, while attention is directed to a secondary task. The completion times for the TUG test and the functional test with the secondary cognitive task are related to measures of cognitive function, particularly executive function [28].

Initially, participants were requested to perform the TUG test without the DT to ensure they understood the motor task correctly. For this test, participants are instructed to rise from a seated position, walk 3 m to a cone, turn around, and return to the starting chair. The time taken to complete the test is recorded. This test was based on those performed in previous studies [29, 30, 31].

For the DT test, they are asked to verbally list all the animals that come to mind during the process. The following instructions were given: ‘When I say GO, please walk as quickly and safely as possible, without running. You need to reach the cone and return to the chair (pointing to the cone and chair). Simultaneously, try to name as many different animals as you can think of, out loud. Remember to do this out loud. It’s important not to stop walking or talking.’

### Background characteristics

Body composition and anthropometric measurements were taken as follows. A 2.10 m portable stadiometer with a margin of error of 0.001 m (SECA, Hamburg, Germany) was used to determine the height of the study participants. A portable bioelectrical impedance scale with a maximum capacity of 200 kg and a margin of error of 50 g (TANITA BC 418-MA, Tanita Corp., Tokyo, Japan) was used to weigh the participants and to obtain other body composition data, such as fat mass or fat-free mass. Participants were measured in light clothing and barefoot.

The Barthel Index was used to assess the subjects’ dependence level. This questionnaire analyses ten basic activities of daily living: feeding, bathing, grooming, dressing, bowel control, bladder control, toilet use, transfers, mobility, and stairs. The score obtained on this scale can range from 0 to 100, where 100 is total independence. A score ≥ 60 points indicates mild dependence, while a score of 40 to 55 points suggests moderate dependence [32].

The Lawton and Brody (L-B) Instrumental Activities for Daily Living Scale was used to asses the ability of individuals to perform tasks necessary for independent living. This tool evaluates more complex activities than basic daily living tasks and is often used to assess older adults. The scale includes eight domains: telephone use, shopping, food preparation, housekeeping, laundry, transportation, medication management and handling finances [33]. It is scored as follows: 0–1 indicates total dependence, 2–3 indicates important dependence, 4–5 indicates moderate dependence, 6–7 indicates light dependence, and 8 indicates independence.

The Mini Nutritional Assessment (MNA) was used to describe the nutritional status of the participants at baseline, as it is closely related to frailty and other age-disease conditions such as sarcopenia [34]. It consists of fifteen questions that gather information about diet, nutritional status, health, and functionality. The score includes anthropometric measures such as BMI, calf circumference, and mid-arm circumference. A score ≥ 24 points indicates that the older adults are well-nourished [35, 36].

### Statistical analyses

In the present study, statistical analyses were performed using IBM SPSS Statistics software (v.25.0 for MAC; IBM Corporation Chicago IL). The normality of the sample was assessed using Shapiro-Wilk tests, and the level of statistical significance was set at *p* < 0.05. Descriptive characteristics were analysed using t-student tests and chi-square tests according to the nature of the variable. Since the participants’ sex did not interact with any of the variables analysed, all participants were treated as a single group, with comparisons made between the CG and the IG. Depending on the nature of the variable, data were presented either as the mean and standard deviation or as the number of participants and the corresponding percentage.

Additionally, repeated measures ANOVA was used to calculate the differences within and between the CG and IG, as well as the group by time interaction, considering the three different evaluation times (before the start of MCT, after completing the 6 months of the intervention and at the end of the 4-month detraining period). Initial analyses were performed without covariates and were then repeated, adjusting for participants’ degree of dependence as measured by the Barthel Index score. This analysis included only those individuals who did not drop out of the study and participated in all three evaluations.

## Results

The main characteristics of the sample are detailed in Table 2. The sample consisted of 108 subjects with a mean age of 80.6 ± 5.9 years, predominantly females (71.3% of the total sample). The present sample were divided into a CG (initially consisting of 55 subjects) and IG (initially consisting of 53 subjects). The minimum attendance registered was 65% of the training sessions.

Initially, only Barthel Index showed significant differences at the beginning of the study (*p* < 0.05), showing a higher score for the individuals of the IG. Cognitive assessment and functional capacity were similar between both groups at baseline (*p* > 0.05).

**Table 2:** Descriptive characteristics of the sample

	Whole sample (*n* = 108)	CG (*n* = 55)	IG (*n* = 53)	*p*-value
Age, mean (SD), years	80.6 (5.9)	80.3 (5.7)	81.0(6.1)	0.565
Sex, n(%)				0.236
Females	77 (71.3)	42 (76.4)	35 (66.0)	
Males	31 (28.7)	13 (23.6)	18 (34.0)	
BMI, mean (SD), kg/m^2^	29.8 (5.61)	29.2 (6.1)	30.3 (5.1)	0.379
Barthel Index mean (SD), points	94.8 (8.5)	93.1 (10.5)	96.6 (5.2)	0.037
L-B, mean (SD), points	10.4(4.3)	10.6(4.3)	10.2(4.3)	0.686
SPPB, mean (SD), points	7.4 (1.5)	7.4 (1.6)	7.5(1.5)	0.856
Prefrail, n(%)	78 (72.2)	40 (72.7)	38 (71.7)	
Frail, n(%)	30 (27.8)	15 (27.3)	15 (28.3)	
MNA, mean (SD), points	24.1 (3.6)	23.7 (4.2)	24.5 (2.9)	0.276
MMSE, mean (SD), points	26.0(3.1)	26.3 (2.9)	26.3 (3.2)	0.989
TUG+DT, mean (SD), s	14.4 (6.9)	15 (6.9)	13.8 (6.9)	0.381

CG: control group; IG: Intervention Group; SD: Standard Deviation; SPPB: Short Physical Performance Battery; MNA: Mini Nutritional Assessment; L-B: Lawton and Brody scale, MMSE: Mini Mental State Examination; TUG+DT: Time up and Go Test with Dual Task.Statistically significant difference was set at *p* < 0.05

Table 3 shows the effects after the 6-month training and 4-month detraining periods. A group-by-time interaction was observed for DT (F(2,62) = 7.675) (*p* > 0.05). Statistically significant changes were observed in the intervention group, with a large effect size (F(2, 58) = 17.509) (*p* < 0.001). Specifically, there was a significant decrease in time spent performing the DT test after the training period, an increase in time during the detraining period, and an improvement when comparing baseline to the end of the detraining period (all *p* < 0.05). Additionally, a difference was observed between the intervention and control groups in the post-training evaluation (F(1,59)) = 4.336, *p* < 0.05.

The CG showed no statistically significant changes during either the 6-month MCT or the detraining period (*p* > 0.05).

No significant changes were found in either group’s MMSE scores or the L-B scale during the training program or the detraining phase (*p* > 0.05).

Moreover, when analyses were repeated with adjustments for the Barthel Index, the group-by-time interaction and within-group comparisons remained statistically significant.

**Table 3:** Effects of a 6-month MCT and a 4-month detraining period within and between control and intervention groups

		Group Effect						Time Effect*							GxT Effect
		Baseline (M0)		Postraining (M6)		Detraining (M10)		M0 to M6		M6 to M10		M0 to M10			
Group	N	Mean (SD)	p (ηp^2^)	Mean (SD)	p (ηp^2^)	Mean (SD)	p (ηp^2^)	Difference (95% CI)	p	Difference (95% CI)	p	Difference (95% CI)	p	p (ηp^2^)	p (ηp^2^)
**TUG+DT**,** s**															
IG	40	13.9 (7.5)	0.438 (0.010)	9.8 (4.5)	0.042 (0.068)	11.0 (5.7)	0.446 (0.010)	4.0 (2.2, 5.7)	<0.001	-1.1 (-2.1,-0,2)	0.010	2.9 (1.0, 4.6)	0.001	<0.001 (0.376)	0.003 (0.108)
CG	21	12.5 (4.4)		12.7 (5.8)		12.2 (5.2)		-0.2 (-2.6, 2.2)	1.000	0.5 (-0.8, 1.8)	0.991	0.3 (-2.2, 2.8)	1.000	0.625 (0.016)	
**MMSE**,** points**															
IG	36	27.0 (2.9)	0.635 (0.005)	27.2 (3.4)	0.854 (0.001)	26.7 (4.2)	0.948 (0.000)	-0.2 (-1.2, 0.8)	1.000	0.6 (-0.7, 1.8)	1.000	0.4 (-0.8, 1.5)	1.000	0.537 (0.027)	0.246 (0.029)
CG	17	27.4 (2.4)		227.4 (3.5)		26.8 (3.9)		0.0 (-1.4, 1.4)	1.000	0.7 (-1.1, 2.5)	0.787	0.7 (-1.0, 2.4)	0.942	0.574 (0.024)	
**L-B**,** points**															
IG	37	9.8 (4.2)	0.989 (0.000)	9.9 (3.0)	0.582 (0.005)	10.2 (4.3)	0.608 (0.004)	-0.1 (-1.8, 1.6)	1.000	-0.3 (-1.2, 0.6)	1.000	-0.4 (-1.9, 1.1)	1.000	0.625 (0.115)	0.246 (0.025)
CG	21	10.3 (4.2)		11.1 (4.0)		9.8 (2.8)		-0.8 (-3.1, 1.4)	1.000	1.3 (0.1, 2.6)	0.029	0.5 (-1.5, 2.6)	1.000	0.034 (0.017)	
**Barthel**,** points**															
IG	41	96.3 (5.4)	0.074 (0.052)	97.3 (6.9)	0.066 (0.055)	97.6 (7.3)	0.294 (0.018)	-1.0 (-3.8, 1.9)	0.172	-0.2 (-2.6, 2.1)	1.000	-1.2 (4.0, 1.5)	0.838	0.550 (0.020)	0.576 (0.009)
CG	22	92.5 (11.4)		93.6 (8.3)		95.5 (8.0)		-1.1 (-5.0, 2.7)	1.000	-1.8 (-5.0, 1.4)	0.503	-3.0 (-5.0, 2.7)	0.172	0.134 (0.065)	

MCT: multicomponent training; M0: Month 0, at baseline; M6: month 6, after 6-month training; M10: month 10 after the 4-month detraining; CG: control group; IG: intervention group; MMSE: Mini Mental State Examination; L-B: Lawton and Brody scale; TUG-DT: Time Up and Go test with Dual Task; GxT: group by time interaction; *pairwise comparisons adjusted by Bonferroni

## Discussion

The primary outcomes of this study reveal three key findings. First, our MCT significantly enhances both functional and cognitive capacities in pre-frail and frail older adults, as demonstrated by improvements in DT performance. Second, a four-month detraining period markedly reduces the gains achieved in DT performance, underscoring the need for continuous engagement in physical activity to sustain these benefits. Third, isolated assessments of mental status and daily living activities did not show significant training effects in frail and pre-frail older adults without cognitive impairment. These results suggest that DT assessments are more sensitive in detecting simultaneous improvements in cognitive and motor performance than isolated functional or cognitive questionnaires.

DT assessments detect subtle deficits in motor and cognitive function that may not be apparent on single-task tests, providing a more comprehensive evaluation of fall risk and other functional and cognitive impairments. Previous research has demonstrated cognitive benefits associated with aerobic and endurance exercise [37]. MCT is widely recognized as the most effective form of exercise for enhancing functional capacity, particularly in frail and pre-frail older adults [10]. More recently, a systematic review and meta-analysis of RCTs have also confirmed its positive effects on cognitive function [38]. However, most studies evaluating cognition have relied on standard tests such as the MMSE, the Montreal Cognitive Assessment, or the Trail Making Test. Given the well-documented effects of MCT on physical function in frail individuals [10], our study’s observed improvements in DT performance could be expected. Nonetheless, research on the effects of MCT on DT performance remains scarce, particularly in frail populations.

The heterogeneity in exercise protocols, participant characteristics, and underlying conditions in this population complicates the ability to draw firm conclusions. The existing literature presents conflicting findings. Ansai and Rebelatto [39] reported smaller improvements in DT performance following MCT than resistance training, potentially due to low adherence. Similarly, Makizako et al. [40] found no significant improvements in DT performance after a six-month MCT intervention, possibly because their sample included individuals with amnestic mild cognitive impairment, suggesting that MCT alone may not be sufficient once cognitive decline has begun. Additionally, including cognitive stimulation at the end of the MCT sessions in our study may have contributed to the observed cognitive benefits.

Conversely, a study involving long-term nursing home residents found that combining cognitive training with MCT did not significantly improve DT performance, likely due to the participants’ severe cognitive and functional impairments [41]. In contrast, Eggenberger et al. [42], reported improvements in both physical exercises alone and in sessions combining physical and cognitive exercises, with slightly greater benefits observed in the combined training group among older adults without cognitive impairment. However, their training program included dance-based video games and treadmill walking, with cognitive training sessions held on separate days. These differences highlight the need for further research to determine the optimal approach to integrating cognitive training or cognitive stimulation with MCT and to establish the most effective dosage based on cognitive status. Some evidence suggests that simultaneous exercise and cognitive training may be more beneficial, as more studies have reported improvements in at least one cognitive domain when both elements are combined [43]. Although systematic reviews on this topic remain limited, MCT appears to be effective in improving DT performance in older adults without cognitive impairment [12], as observed in our study, where a short period of cognitive stimulation followed the physical training. However, its effects may be less pronounced in healthy, robust individuals due to their more advanced motor skills. All these results suggest that MCT with cognitive stimulation is particularly beneficial for individuals at risk of frailty or functional decline without including additional cognitive training sessions.

The lack of significant findings in separate analyses of our study’s mental status and daily life activities may be attributed to several factors, including subjective perceptions of daily tasks, ceiling effects associated with the MMSE, and sample characteristics. Despite being at risk of frailty, our participants did not exhibit cognitive impairments severe enough to impact daily activities, which may explain the absence of measurable improvements in these domains—consistent with previous research [44]. Nevertheless, the observed enhancements in DT performance suggest a potential reduction in fall risk [5] and may also serve as a preventive measure against future cognitive decline [45]. However, this hypothesis needs to be tested in future studies.

This study is among the few that examine not only the immediate benefits of exercise but also the effects of detraining, offering a comprehensive perspective on the long-term impact of MCT on DT performance in frail and pre-frail older adults. Few studies have explored this area; for example, two previous studies assessed changes following a detraining period. In contrast to our findings, Ansai and Rebelatto [39] reported no decline in DT performance after three months of detraining, though this may be attributed to the smaller initial gains and low adherence during their four-month training period. Meanwhile, our Elder-fit program demonstrated high adherence, which may explain the differences in outcomes. Additionally, research on healthy older adults has shown that DT performance did not decline even after one year of detraining, though they exhibited improvements immediately after training [42]. This contrasts with our results, where we observed significant decreases in DT performance following detraining, although performance did not fully return to baseline levels. These findings suggest that well-structured MCT interventions may sustain DT-related benefits longer than general fitness programs alone [46]. Our standardized training protocol provides a valuable reference point and contributes to the existing body of evidence with a clear and replicable model.

### Strengths and limitations

Several limitations warrant mention. Firstly, the non-randomized study design, while intended to minimize participant dropout and maintain consistency throughout the study, led to fewer participants completing all three main assessments, particularly in the CG. Although no baseline differences were observed in the SPPB, the lower Barthel Index score in the CG may have influenced the results. Nevertheless, the intervention was rigorously structured, with standardized procedures and the use of validated questionnaires. However, ceiling effects could limit its sensitivity to detect improvements in the MMSE and both functional questionnaires. Lastly, as it is a composite measure, the TUG has certain limitations, including a potential floor effect and challenges in distinguishing gait components within the total score. Additionally, its reliability has not been thoroughly investigated.

## Conclusion

MCT enhances both functional and cognitive capacities through DT performance in pre-frail and frail older adults without cognitive impairments. However, detraining periods of at least four months result in a decline in these benefits, highlighting the need for continuous physical activity to sustain functional and cognitive improvements. To mitigate the effects of detraining, it is essential to promote ongoing engagement in physical exercise, even at reduced intensity. Future research should prioritize developing tailored exercise programs that incorporate strategies to minimize the impact of training interruptions. Potential solutions include flexible home-based activities or video-assisted training sessions that are easy to sustain. These approaches are crucial for designing effective interventions that support long-term health and independence in the aging population and merit further investigation.

## Data Availability

The data supporting this study’s findings are available from the GENUD Research Group. However, restrictions apply to their availability. The data were used under licence for the current study and are not publicly available. However, they are available from the authors upon reasonable request.
